# Music Restores Propriospinal Excitation During Stroke Locomotion

**DOI:** 10.3389/fnsys.2020.00017

**Published:** 2020-04-09

**Authors:** Iseline Peyre, Berthe Hanna-Boutros, Alexandra Lackmy-Vallee, Claire Kemlin, Eléonore Bayen, Pascale Pradat-Diehl, Véronique Marchand-Pauvert

**Affiliations:** ^1^Sorbonne Université, Inserm, CNRS, Laboratoire d'Imagerie Biomédicale, LIB, Paris, France; ^2^Sorbonne Université, CNRS, Institut de Recherche et de Coordination en Acoustique Musique (IRCAM), UMR Sciences et Technologies de la Musique et du Son (STMS), Paris, France; ^3^Physical Therapy Department, Holy Family University, Batroun, Lebanon; ^4^Sorbonne Université, AP-HP, GRC n°24, Handicap Moteur et Cognitif & Réadaptation (HaMCRe), Paris, France

**Keywords:** propriospinal neurons, spinal cord, locomotion, stroke, music therapy

## Abstract

Music-based therapy for rehabilitation induces neuromodulation at the brain level and improves the functional recovery. In line with this, musical rhythmicity improves post-stroke gait. Moreover, an external distractor also helps stroke patients to improve locomotion. We raised the question whether music with irregular tempo (arrhythmic music), and its possible influence on attention would induce neuromodulation and improve the post-stroke gait. We tested music-induced neuromodulation at the level of a propriospinal reflex, known to be particularly involved in the control of stabilized locomotion; after stroke, the reflex is enhanced on the hemiparetic side. The study was conducted in 12 post-stroke patients and 12 controls. Quadriceps EMG was conditioned by electrical stimulation of the common peroneal nerve, which produces a biphasic facilitation on EMG, reflecting the level of activity of the propriospinal reflex between ankle dorsiflexors and quadriceps (CPQ reflex). The CPQ reflex was tested during treadmill locomotion at the preferred speed of each individual, in 3 conditions randomly alternated: without music vs. 2 arrhythmic music tracks, including a pleasant melody and unpleasant aleatory electronic sounds (AES); biomechanical and physiological parameters were also investigated. The CPQ reflex was significantly larger in patients during walking without sound, compared to controls. During walking with music, irrespective of the theme, there was no more difference between groups. In controls, music had no influence on the size of CPQ reflex. In patients, CPQ reflex was significantly larger during walking without sound than when listening to the melody or AES. No significant differences have been revealed concerning the biomechanical and the physiological parameters in both groups. Arrhythmic music listening modulates the spinal excitability during post-stroke walking, restoring the CPQ reflex activity to normality. The plasticity was not accompanied by any clear improvement of gait parameters, but the patients reported to prefer walking with music than without. The role of music as external focus of attention is discussed. This study has shown that music can modulate propriospinal neural network particularly involved in the gait control during the first training session. It is speculated that repetition may help to consolidate plasticity and would contribute to gait recovery after stroke.

## Introduction

Stroke is the third main cause of disability in the world (World Health Organization, 2014). The after-effects include multiple sensory, cognitive and motor impairments, which limit the autonomy in the daily life activities at different degrees from one individual to another (Cerniauskaite et al., 2012). In particular, the gait recovery represents one of the major emphasis during rehabilitation (Wade et al., 1985). Among the various rehabilitation approaches, programs based on the use of sounds and music are the subject of a growing interest. With the emergence of music therapy profession, several novel sound or music-based methods and interventions for rehabilitation have been developed, including the use of rhythmic auditory stimulation (RAS), music listening, music practice (instrumental or vocal) and, more recently, the use of device-assisted real-time sound-movement coupling (movement sonification). Short-term benefits of RAS on gait parameters, including gait freezing, and on non-motor functions (mood, anxiety) have been reported especially in Parkinson disease, and the preliminary results in other movement disorders are promising (Burt et al., 2019; Devlin et al., 2019). In patients with stroke, several studies on music-supported therapies during rehabilitation of upper limb have reported an improvement of motor functions, together with changes in neural excitability and connectivity at the brain level (Altenmüller et al., 2009; Schneider et al., 2010; Rojo et al., 2011; Amengual et al., 2013; Grau-Sánchez et al., 2013; Ripollés et al., 2016; Ghai, 2018). Regarding the locomotor functions, the approach most commonly tested so far used RAS, which consists in beating a regular tempo with a metronome calibrated on the steps of the patients. The step synchronization with the metronome improves the cadence, the step length and the walking speed (Yoo and Kim, 2016), and a Cochrane review has concluded in favor of the potential benefits of this method to improve gait after stroke (Magee et al., 2017).

Beyond the effects on the locomotor rhythmicity, the patients may pay attention to the sound or to the music they are listening to, which might also influence their walking abilities. In line with this, it has been shown that internal focus of attention during post-stroke gait rehabilitation, e.g., encouraging the patients to be aware of their movements and their performance, may reduce automaticity and hinder learning and retention (Johnson et al., 2013). Inversely, an external focus of attention, e.g., asking the patients to walk on markers on the floor, is useful to improve post-stroke walking (Kim et al., 2019). In healthy subjects, the attention paid to movement modulates the activity in the sensory-motor brain areas, and different subareas in the motor cortex are activated during attended and unattended movements (Binkofski et al., 2002; Johansen-Berg and Matthews, 2002). After stroke, the attention on movement is reinforced, even for stereotyped movement like locomotion. Therefore, there is a possibility that the neural networks for attended and unattended movements are modified after stroke, which would reflect on descending inflow, modulating the subcortical excitability, including the one of spinal cord. Our first objective was to distract the patients and to draw their attention away from their walk, but not too much to limit the risk of falls which is increased when stroke patients are too much disturbed by external cues (external distractors). To test the hypothesis whether music would keep the attention of the patients and would influence post-stroke locomotion independently of the well-known effect of rhythm cues on the locomotor cadence, we tested the effect of music with irregular tempo (arrhythmic music), one pleasant and one unpleasant assuming that both would captivate the patients differentially.

It has been well-established that listening to music and its practice induce remodeling of brain structures, with reorganization of neural networks in both healthy subjects and stroke patients (Altenmüller and Schlaug, 2015; O'Kelly et al., 2016). Regarding the spinal cord, which is particularly involved in the control of locomotion (Grillner and El Manira, 2019), the effect of sound on spinal activity has started to be explored at the beginning of the 20th century. In 1914, Forbes and Sherrington have observed a facilitating effect of sound on the Hoffmann (H) reflex in decerebrate cats (Barnes and Thomas, 1968). Later on, the neuromodulatory effect of sound on spinal reflexes has also been reported in humans (Rossignol and Jones, 1976; Delwaide and Schepens, 1995; Ruscheweyh et al., 2011; Roy et al., 2012). To date, no study has explored the effect of music on spinal excitability during post-stroke walking. Accordingly, in the present study, we have addressed the question whether a pleasant and unpleasant arrhythmic music would modify differentially the spinal cord excitability and gait parameters in stroke patients, compared to healthy subjects. The spinal excitability was evaluated by investigating the propriospinal reflex that is particularly involved in the control of lower limb muscle synergies during posture and locomotion in humans (Pierrot-Deseilligny and Burke, 2012). This reflex constitutes an interesting metric of spinal activity during locomotion for evaluating the neuromodulation. Classically, the propriospinal excitation is investigated by studying the modulation of the biphasic facilitation produced by electrical stimulation of the common peroneal (CP) nerve on quadriceps (Q) electromyogram (EMG), termed as CPQ reflex in the following. Accordingly, we investigated the modulation of the CPQ reflex during post-stroke locomotion, to determine whether the music can modify it, independently from its rhythmic effect, but by its possible influence on attention.

## Methods

### Ethical Approval

The study conformed to the latest revision of the Declaration of Helsinki. The procedures were approved by Assistance Publique-Hôpitaux de Paris (AP-HP, clinical research sponsor) and have obtained the authorizations of the national French ethics committees (CPP Ile de France VI—Pitié-Salpêtrière; PHRC 95078, DRCD 070804). All the subjects have provided their written informed consent prior their inclusion in the experimental procedure.

### Inclusion Criteria

Twelve patients with a first history of stroke were included in the study (3 females; mean age ± 1 standard deviation, SD: 55.8 ± 13.5; range 29–78 years old). All but 1 had a unilateral lesion (supported by MRI or CT-scan examination): 9 had right hemiparesis, 2 left hemiparesis and the last one had bilateral lesions but predominant hemiparesis on the right side. The cause of stroke was ischemia in 10/12 patients, and hemorrhage in the 2 last remaining patients. At the time of the experiments, the time since stroke ranged from 2 to 17 months (6.3 ± 5.3 months). All the patients had experienced severe gait impairment lasting more than 1 week after stroke, and 5/12 patients did not use walking aid anymore at the time of the experiment; the 7 other patients used a cane, plus an ankle splint in 2 of them. All of them were able to walk at least 20 min on the treadmill, by holding the security bars (laterally or frontally); the body weight support device was not used in this study. Seven patients exhibited cognitive and mood disorders, including attention deficit in 1, depression in 1, partial anosognosia in 1, apraxia in 2, hemineglect in 1, slight cognitive impairments in 2. Only 5 patients receive botulinum toxin therapy but not in the investigated muscles (tibialis anterior—TA—and quadriceps), and only 1 patient had oral antispastic (dantrolene sodium 50 mg/day; see Table 1).

**Table 1:** Clinical data and CPQ reflex.

						**CPQ**			**Spasticity**			
**Patient**	**Delay**	**Hemiparesis**	**Origin**	**Walking aid**	**Cognitive impairments**	**No music**	**With melody**	**With AES**	**Medication**	**TA**	**Soleus**	**VL**
1	2	R	Isch.	+	–	145.7	134.3	117.6	–	1	1	1
2	5	R+	Isch.	+	+	224.0	211.5	140.3	BoNT	0	1	0
3	9	R	Hem.	+	+	148.6	133.8	113.2	BoNT	0	4	1
4	2	R	Isch.	–	–	213.2	194.5	176.8	Dandro.	0	1	2
5	17	R	Isch.	+	–	134.5	118.7	106.7	BoNT	3	3	2
6	6	R	Hem.	+	–	148.7	121.6	138.7	BoNT	1	4	3
7	16	L	Isch.	–	+	115.6	121.3	85.2	–	2	2	1
8	3	R	Isch.	+	–	142.8	95.4	125.6	BoNT	0	3	2
9	7	R	Isch.	+	+	142.5	111.5	124.4	–	0	0	0
10	3	R	Isch.	–	+	184.0	143.2	143.2	–	0	0	0
11	2	L	Isch.	–	+	274.0	172.5	167.5	–	0	0	0
12	3	R	Isch.	–	+	130.6	109.5	118.6	–	0	0	0

*Patient, Inclusion rank of the patient; Delay, time since stroke in months; Hemiparesis, side of hemiparesis with R for right, R+ for right predominance and L for left; Origin, Isch. for ischemia and Hem. for hemorrhage; Walking aid, patients using canes (+) or not (-); Cognitive impairment, patients with (+) or without cognitive impairments (-); CPQ, size of CPQ reflex (area of conditioned EMG % mean unconditioned EMG), observed during walking without sound, with melody and with AES; Medication, patients with medication for spasticity (Botulinum toxin injection, BoNT, or Dantrolene, Dantro.) or not (-); TA, Soleus; VL, score to rectified Ashworth scale (Spasticity)*.

Twelve healthy subjects (controls) participated in the experiments (4 females; 52.0 ± 6.5 years old; range 32–67 years old). The inclusion criteria included no history of neurological disorders and no orthopedic trauma in lower limbs.

### Recordings

**Figure 1:**
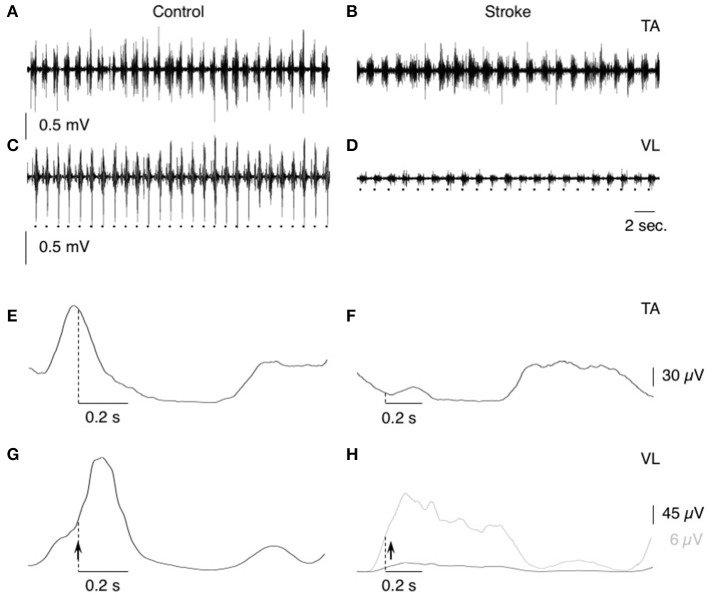
EMG activities during treadmill walking. **(A–D)** Raw EMG activity during treadmill walking in tibialis anterior (TA; **A,B**) and vastus lateralis (VL; **C,D**) in one control (speed 1 m.s^−1^; **A,C**) and one patient (speed 0.33 m.s^−1^; **B,D**). The dots at the bottom indicate the time of heel contact. **(E,F)** the raw EMG activities have been rectified and 50-ms smoothed before averaging (*N* = 50 steps) in tibialis anterior (TA; **E,F**) and vastus lateralis (VL; **G,H**) in the same individuals as in **(A–D)**. In **(H)** the gray line represents the mean EMG activity with optimized Y-scale for the patient. Dotted vertical lines indicate the time of heel contact, and vertical arrows, when the conditioning stimuli were delivered during the gait cycle.

**Figure 2:**
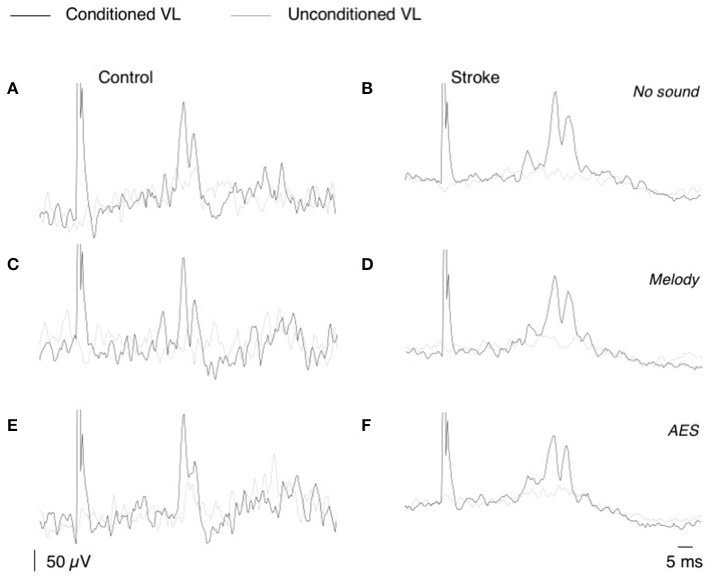
CPQ reflex produced by CP nerve stimuli on VL EMG. **(A,F)** Mean unconditioned (gray line) and mean conditioned (black line) rectified VL EMG in one control subject **(A,C,E)**, and one patient **(B,D,F)**; same subjects as in Figure 1. The CP nerve stimulation (*N* = 50, 2–2.5 × MT) was triggered by the heel contact and was delivered with 0-ms delay in the control subject and 40-ms delay in the patient. Unconditioned and conditioned recordings were randomly alternated during the EMG acquisition performed without sound **(A,B)**, with melody **(C,D)**, or AES **(E,F)**.

EMG activities were recorded using single-use bipolar surface electrodes (foam electrodes with solid gel; 2-cm apart; FIAB, Florence, Italy) that were secured on the skin, over the muscle belly of TA and vastus lateralis (VL). The electrodes were plugged to wifi connectors that transmitted the signals to an EMG zero wire system (Cometa Srl, Milan, Italy). The signals were amplified and filtered (x 1,000–5,000; 10–500-Hz bandpass) before being digitally stored on a personal computer (2-kHz sampling rate; Power 1,401 controlled by Spike2, CED, Cambridge, UK). A pressure transducer was placed under the foot, to time the ground contact and to determine the beginning of the walking stance phase. The transducer was connected to the EMG zero wire system by wifi, to synchronize the recording of EMG activities and the signal from foot contact (Figures 1A–D). The delay for the wifi transmission was 12 ms for both the foot contact and EMG recordings, so we did not need to realign the TTL and EMG signals and the 0-ms latency was centered on the artifact to evaluate the latency of the CPQ reflex (Figure 2). In controls, the transducer was placed on the heel of the foot (in the shoe), and in patients, on the forefoot (middle and external aspect of the plantar aspect of the foot; in the shoe, as in controls). Indeed, the patients did not contact the ground with the heel but with forefoot because of foot drop on the paretic side and slow speed (as in Achache et al., 2010). The patients were tested on their hemiparetic side only, because the CPQ reflex was found particularly enhanced on this side, compared to the non-paretic side (Achache et al., 2010); the controls were tested on the right side.

The recordings were performed during stabilized treadmill locomotion (Biodex Medical Systems Inc., Shirley, New York, USA). At the beginning of the experiment, the subjects walked on the treadmill for 5–10 min before recordings, to accustom themselves to treadmill walking, and to determine their comfortable speed: 0.2 ± 0.1 m.s^−1^ (0.1–0.4) in patients vs. 1.0 ± 0.2 m.s^−1^ (range 0.7–1.3) in controls (*t*-test, *p* < 0.001). In patients, the speed for recordings was not their maximum speed, but it was the one at which they felt secure and they were able to walk for 2–3 min (~ recording duration) without fatigue. The walking speed in each individual was constant throughout the experiment to avoid any change in the CPQ reflex due to speed rate changes (Iglesias et al., 2008b).

### Conditionings

Percutaneous electrical stimulations (1-ms duration rectangular pulse; DS7A, Digitimer Ltd, Hertfordshire, UK) were applied to the CP nerve through bipolar surface electrodes (two 2-cm diameter brass half balls covered by wet sponge tissue, spaced by ~5 cm). The electrodes were placed on each side of the fibula neck, to evoke a motor response first in TA, without activation of the peroneal muscle group at the motor threshold (checked by tendon palpation during quiet standing). The CP nerve stimulation is then more effective for eliciting a biphasic excitation in VL motoneurons (CPQ; Simonetta-Moreau et al., 1999). During walking, the CP nerve stimulations were triggered by a TTL signal generated by the signal from the pressure transducer, at the time of foot contact.

### Experimental Procedures

The first acquisition consisted in collecting EMG activities in TA and VL, and the foot contact signals during walking at comfortable speed in each participant, without conditioning, to design the walking pattern (Figures 1E–H). According to previous studies (Marchand-Pauvert and Nielsen, 2002; Iglesias et al., 2008b), the trigger delay (after foot contact) for the CP nerve stimulation was determined in each individual, according to their walking pattern so as to deliver the conditioning stimuli at the beginning of the walking stance phase, within the first part of the ascending phase of the VL EMG burst, so as to produce the CPQ reflex within the middle of this phase. Only 1 trigger delay was tested in each individual, and it was kept constant throughout the experiment, to avoid any change in CPQ reflex due to a change in the trigger delay (Marchand-Pauvert and Nielsen, 2002). The mean trigger delay was 32.9 ± 23.5 ms (range 0–65 ms) in controls vs. 52.1 ± 11.8 ms (range 0–120 ms) in patients (Mann-Whitney U test, *p* = 0.32). The maximal amplitude of the direct motor (M) response in TA EMG (Mmax) was measured when the subjects walked at the speed and the delay investigated. The stimulus intensity was then reduced so as to produce a constant M response of ~80% of Mmax, whose size was monitored throughout the experiment. This response was produced with stimulus intensities around 2–2.5 x the threshold for M response (x MT), i.e., above the threshold for activating group I and group II muscle spindle afferents (Pierrot-Deseilligny and Burke, 2005). At this intensity, the stimulation did not perturb the gait cycle. During a recording session, 100 foot-contacts triggered the computer, which randomly delivered 50 CP nerve stimulations (conditioned EMG) or no stimulation (unconditioned EMG). Three recording sessions were randomly alternated in each group: 1 without sound, 1 with a melody (Yiruma, 2001), and 1 with aleatory electronic sounds (AES, especially composed for the protocol; Supplementary Audio). At the beginning of each recording session, the subjects walked without music, and when the locomotion was stable, we turned on the music (or not in the no-sound condition), and then the computer, to trigger the stimulations and to record the EMG activities. Music sound was not used as a trigger; it was continuously displayed all during the recording session. The volume of the sound was determined at the beginning of the protocol, covering the treadmill noise, and we made sure that it was comfortable for each individual and it did not induce any startle response (especially when we turned the music on). Both the melody and AES had a variable tempo and were similar for each individual. None of the subjects had already heard the 2 musical themes before the experiment. They listened both themes only during the 2 recording sessions with music. At the end of the experiment, each individual indicated what he thought about each musical theme.

Biomechanical and physiological parameters were collected during the recording sessions, at the same time than EMG activities. The sensors below the belt of the treadmill allows to record each step during the gait cycle. The subjects wore a thoracic belt with sensors that allowed to collect by Bluetooth, the heartbeat and the breathing rhythm (Zephyr^TM^ performance system, Medtronic, Boulder, CO, USA; Labchart, ADInstruments Ltd., Thame Oxfordshire, UK).

### Analysis

The CP nerve stimulation produced a biphasic facilitation on the rectified VL EMG averages (Figure 2), and an area analysis was performed for a quantitative estimate of the corresponding excitation produced in VL motoneurons (CPQ reflex; Marchand-Pauvert and Nielsen, 2002; Achache et al., 2010). For this, the mean conditioned and unconditioned EMG were superimposed to determine the latency and the duration of the CPQ reflex (e.g., 32.5–41.5 ms in the control illustrated in Figures 2A,C,E, and 34–46.5 ms in the patient illustrated in Figures 2B,D,F); the area of the CPQ reflex was evaluated within the analysis window confined to its latency and duration. Both conditioned and unconditioned EMG were analyzed within the same analysis window, and the area of the conditioned EMG was normalized to the mean area of the unconditioned EMG, giving a quantitative metric of the excitation underlying the CPQ reflex. In each individual, the same window of analysis was used for analyzing the EMG collected during the session without sound and those during the 2 sessions with music.

The ambulation index was automatically calculated by the software of the treadmill. This index is a composite score relative to 100, based on foot-to-foot time distribution ratio and average step cycle. The score 100 indicates that the walking pattern was symmetrical (step length and duration, duration of single and double support phase); the smaller the index the less symmetrical the gait.

### Statistics

Statistical analyses were performed with SigmaPlot 13.0 (Systat Software Inc., San Jose, CA, USA). The significance level α was fixed at 0.05 and the results were considered statistically significant only if *p* < 0.05. Mean values are indicated ± 1 standard deviation. Homoscedasticity (Levene median test) and normality (Shapiro-Wilk test) were first verified to allow parametric analyses (unpaired *t*-test, two-ways ANOVA). Alternatively, non-parametric methods were used (Mann-Whitney U test, ANOVA on ranks), and *post hoc* multiple pairwise comparisons were performed with Student Newman Keuls method. For clarity, the statistical tests and the parameters included in each test are specifically indicated in Results.

## Results

### Music Induced CPQ Reflex Modulation During Post-stroke Locomotion

**Figure 3:**
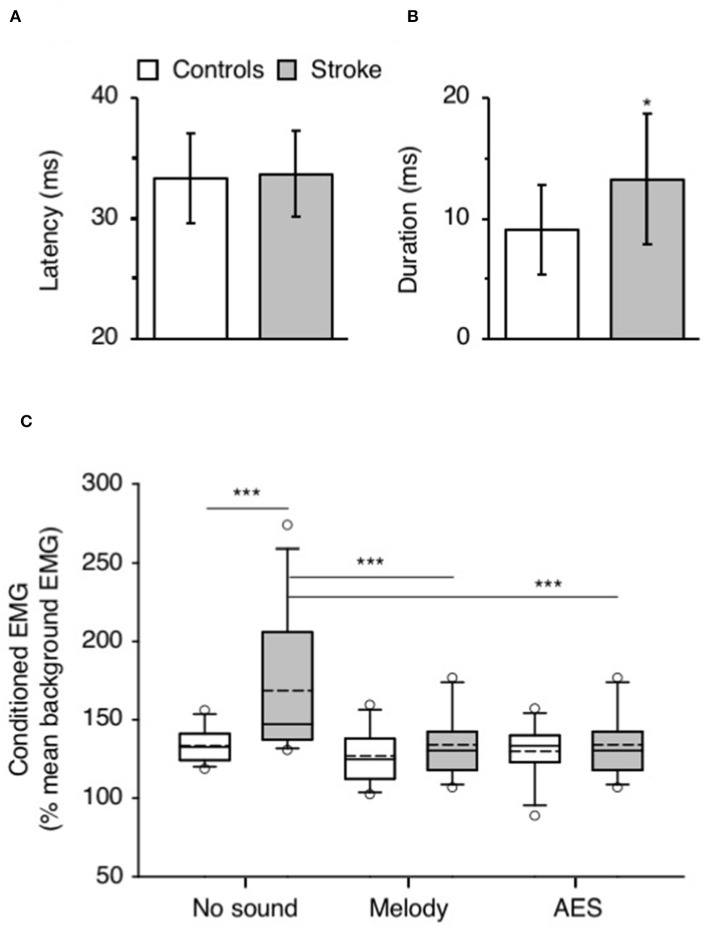
Music-induced modulation of CPQ reflex. **(A)** Mean latency (ms) of CPQ reflex in controls (white column) and in patients with stroke (gray column). The vertical error bars indicate ± 1 × SD. **(B)** Mean duration (ms) of the CPQ reflex in both groups, same legend as in **(A)**. **(C)** Mean CPQ reflex expressed as the area of conditioned EMG (% mean unconditioned EMG) in the group of controls (white box; *N* = 12) and in the group of stroke patients (gray box; *N* = 12). The CPQ reflex was investigated during walking without sound and when listening to melody or AES. The box plot charts illustrate the data distribution in each condition. The boundary of the box closest to 0 indicates the 25th percentile (Q1), the continuous line within the box marks the median and the dotted line, the mean. The boundary farthest from 0 indicates the 75th percentile (Q3). The whiskers (error bars) above and below indicate the 90th and 10th percentiles, respectively. The open circles represent the 5% outliers. *p < 0.05, ***p < 0.001.

Figure 2 shows that CP nerve stimuli produced a biphasic facilitation on the mean rectified VL EMG (CPQ reflex) at a latency about 30–35 ms after the stimulus, in both the control (Figures 2A,C,E) and the patient with stroke (Figures 2B,D,F). A CPQ reflex was similarly observed in VL EMG in all the participants. Figure 3A illustrates the mean latency of the CPQ reflex in controls and patients, which occurred with similar latency in both groups (33.6 ± 3.7 ms in controls vs. 33.7 ± 3.5 in stroke; *t*-test, *p* = 0.97). On the other hand, the duration of the CPQ reflex was significantly longer in patients than in controls (13.2 ± 5.4 vs. 9.0 ± 3.7 ms; *t*-test, *p* < 0.05).

In Figure 2, the area of the CPQ reflex was larger in the patient than in the control (compare the difference between black and gray lines). In the control, the CPQ reflex was hardly modified with music while in the patient, both the first and the second peak of the CPQ reflex (early and late component) were smaller with music, compared to no-sound condition. In the group of patients, the 2 components were similarly modified with music. Therefore, and to facilitate the reading, we only present the modulation of the CPQ reflex in both groups, without dissociating the early and late component. The box plots in Figure 3C illustrate the size range of the CPQ reflex in the group of controls and the group of patients. ANOVA on ranks was significant (*p* < 0.05), and *post hoc* pairwise comparisons (Student Newman Keuls method) revealed that the CPQ reflex was significantly larger in patients than in controls during walking without sound (*p* < 0.001); during walking with music, irrespective of musical theme, there was no more difference between groups. In the group of controls, the CPQ reflex was similar, whether the subjects listened to music or not (0.78 < *p* < 1). In the group of patients, the CPQ reflex was significantly larger during walking without sound (no sound vs. melody, *p* < 0.001; no sound vs. AES, *p* < 0.001), and it was of similar size, whether the patients listened to the melody or to AES (melody vs. AES, *p* = 0.78). The music-induced changes in the CPQ reflex size were rather homogeneous across the patient group: the CPQ reflex was depressed when listening to music in all patients, except in the patient #7 in whom it was depressed only when listening to AES (Table 1).

Walking speed, trigger delay for CP nerve stimuli and background EMG influence the CPQ size during walking (Marchand-Pauvert and Nielsen, 2002; Iglesias et al., 2008b). Both walking speed and trigger delay were kept constant throughout the experiment. Two-ways ANOVA was performed to compare the background EMG, and as could be expected given the lower speed in patients, compared to controls, the mean level of unconditioned EMG was lower in patients than in controls (*p* < 0.001) but it did not change between trials without sound and those with melody or AES (*p* = 0.18; interaction subject group x sound context: *p* = 0.14; Table 2). Therefore, the change in CPQ reflex within a group cannot be supported by a change in the level of the background EMG when listening to music or not.

**Table 2:** Background EMG. Mean unconditioned EMG (mV/ms; ± 1 SD) in controls (1st raw) and patients with stroke (2nd raw) during walking without sound (1st column), when listening to melody (2nd column), or to AES (3rd column).

	**No sound**	**Melody**	**AES**
**Controls**	0.315 ± 0.171	0.370 ± 0.245	0.333 ± 0.213
Stroke^***^	0.079 ± 0.055	0.082 ± 0.057	0.079 ± 0.056

*** *p < 0.001*.

### Sound Preferences

At the end of the experiment, the participants gave their impression on the musical themes, whether they preferred one or another. All of them but 1 preferred the melody, compared to AES; 1 control preferred AES. Another control reported that AES were annoying, and another one indicated that he found them very strange. Most of the patients preferred to walk with music than without, and they preferred the melody. Three patients have indicated that they found AES annoying and strange, and were particularly attentive to it. However, they found the melody was more pleasant, rocked by it.

### Biomechanical and Physiological Parameters

**Figure 4:**
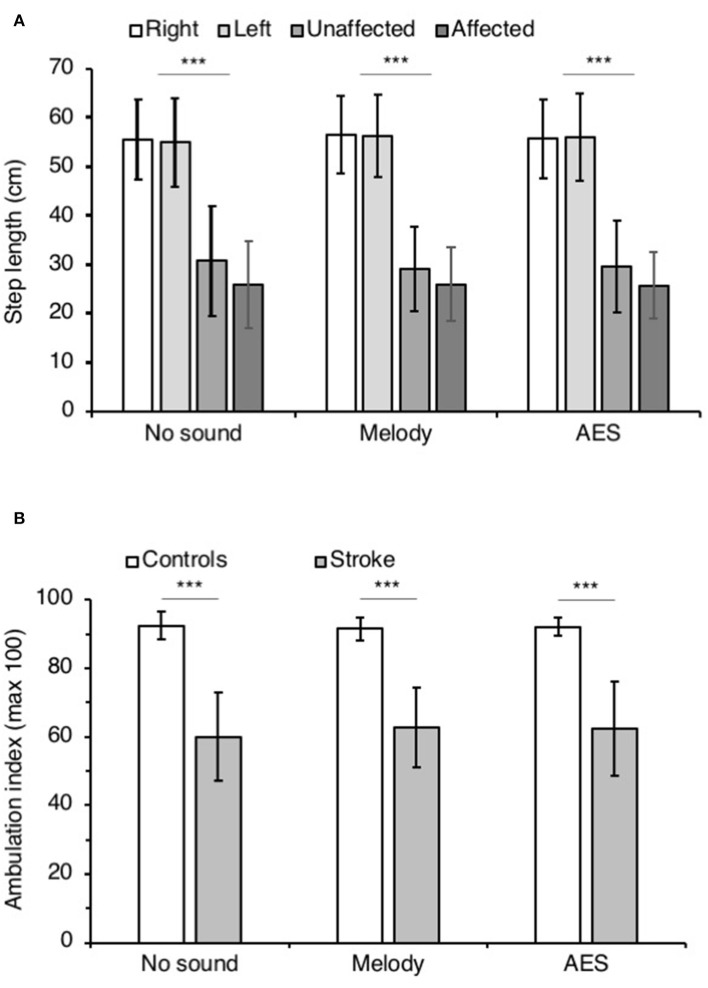
Step parameters. **(A)** Mean step length (cm) in controls on the right (white columns) and the left side (light gray), and in patients with stroke on the affected (middle gray columns) and the unaffected side (dark gray columns). The vertical error bars indicate ± 1 × SD. **(B)** Mean ambulation index in controls (white columns) and in patients with stroke (gray columns). Vertical bars as in **(A)**. The step parameters were collected during walking without sound and when listening to melody or AES. ***p < 0.001.

Figure 4A shows the mean step length on both sides in controls and patients. ANOVA on ranks was significant (*p* < 0.001), and *post hoc* pairwise comparisons (Student Newman Keuls method) revealed significant longer step length in controls than in patients (*P* < 0.001), whatever the audio conditions. In patients, the step length tended to be smaller on the affected side but the difference was not significant (0.10 < *p* < 0.93). Most importantly, the music had no influence of the step length in both groups (0.35 < *p* < 0.99 in controls, 0.10 < *p* < 0.93 in stroke patients).

Figure 4B illustrates the mean ambulatory index in both groups. Irrespective of the audio context, the ambulation index was significantly smaller in patients than in controls (ANOVA on ranks *p* < 0.001; Student Newman Keuls *p* < 0.001 for all comparisons controls vs. stroke). In both groups, the ambulation index did not change whether the participants listened to music or not (0.52 < *p* < 0.73 in controls, and 0.56 < *p* < 0.99 in patients).

**Figure 5:**
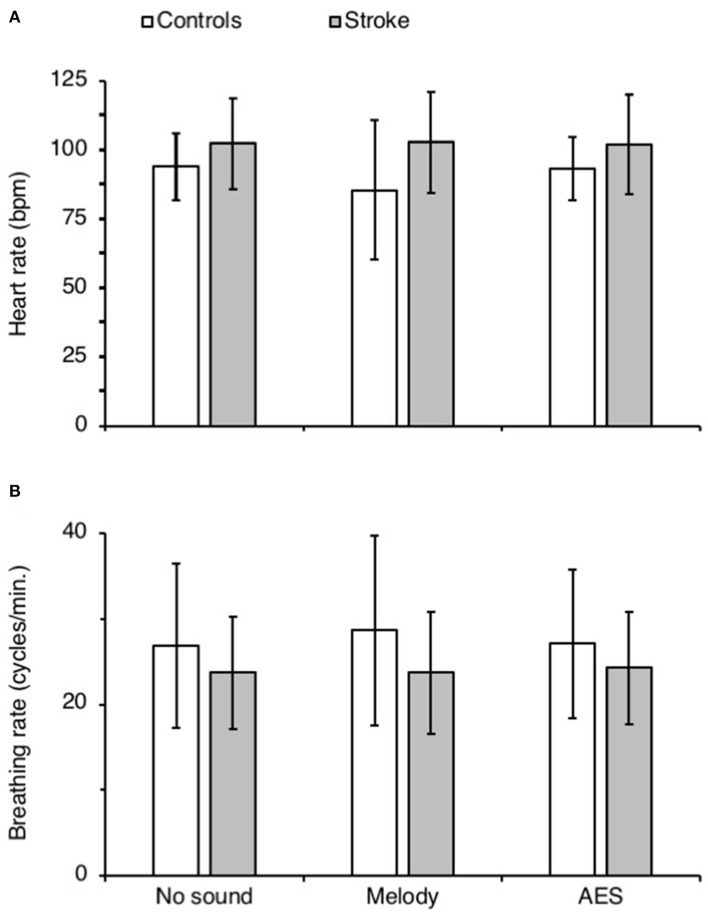
Physiological parameters. **(A,B)** Mean heart (bpm, **A**) and breathing rate (cycles.min.^−1^) in controls (white columns) and in patients with stroke (gray columns), calculated during each recording session with (Melody or AES) and without music (No sound). The vertical error bars indicate ± 1 × SD.

Figure 5 shows the mean heart rate (Figure 5A) and the mean breathing rate (Figure 5B) in both groups, in each audio context. ANOVA on ranks did not reveal any difference between groups and audio contexts for both physiological parameters (*p* = 0.28 for heart rate and *p* = 0.87 for breathing rate).

## Discussion

This study has shown that the CPQ reflex, particularly enhanced on the paretic side of post-stroke patients, was significantly depressed when listening to a music theme, whether a melody or AES, during walking. With music, the CPQ reflex in patients recovered a normal size, as observed in matched controls. On the other hand, music had no influence on the CPQ reflex in controls. Lastly, the music-induced neuromodulation was not accompanied by any change in biomechanical and physiological parameters.

### Origin and Role of the CPQ Reflex

The CPQ is mediated by group I and group II muscle spindle afferents from ankle dorsiflexors, which project onto spinal interneurons impinging on motoneurons supplying quadriceps (Forget et al., 1989a,b; Marque et al., 1996; Chaix et al., 1997). Several lines of evidence support that the interneurons transmitting the excitation to quadriceps motoneurons in humans, are similar to the L3-L4 mid-lumbar interneurons in cats, also termed as propriospinal neurons or group II interneurons (Jankowska, 1992; Pierrot-Deseilligny and Burke, 2005). These interneurons are part of a complex spinal neural network that constitutes a crossroad between the motor cortex, the midbrain monoaminergic structures, and the peripheral mechanoreceptors. The descending (of pyramidal and extrapyramidal origin) and peripheral inputs (of various origins) are integrated by the lumbar propriospinal system that does not only transmit the motor command to motoneurons passively, but integrates all the converging inputs and diffuses its projections onto lumbar motoneurons (both agonists and antagonists), for a fine control of the lower limb muscle synergies during movement (Chaix et al., 1997; Marchand-Pauvert et al., 1999; Simonetta-Moreau et al., 1999; Rémy-Néris et al., 2003; Maupas et al., 2004; Pierrot-Deseilligny and Burke, 2005). It has been shown that the lumbar propriospinal system is particularly involved in the control of posture and locomotion in humans (Marchand-Pauvert and Nielsen, 2002; Marchand-Pauvert et al., 2005). More importantly, the propriospinal reflex between ankle dorsiflexors and quadriceps, is particularly enhanced during walking at the preferred speed (more automatic than slow or fast speed walking), independently of motor cortex influence, which led the authors to propose that this reflex is likely involved in the automatic control of posture during locomotion, assisting the contraction of quadriceps during the lengthening contraction of ankle dorsiflexors, at the beginning of stance (Marchand-Pauvert and Nielsen, 2002; Marchand-Pauvert et al., 2005; Iglesias et al., 2008a,b, 2012). After stroke, the propriospinal excitation has been found particularly enhanced on the paretic side of patients, at rest and during walking (Marque et al., 2001; Achache et al., 2010). The late part of the CPQ reflex (mediated by group II afferents) is particularly enhanced during post-stroke walking, whether the patients are compared to controls walking at their preferred speed (~1.0 m.s^−1^) or slowly (at ~0.2 m.s^−1^ like stroke patients; Figure 3E in Achache et al., 2010). The CPQ reflex increased with the level of background EMG (Marchand-Pauvert and Nielsen, 2002), which could participate in the difference in reflex size between controls and patients but this is unlikely because (i) the level of background EMG was lower in patients (especially due to slow speed) than in controls, so a smaller CPQ reflex could be expected instead, and (ii) Achache et al. (2010) reported smaller CPQ reflex in controls walking at the same speed and with similar background EMG as in patients. To our knowledge, the propriospinal reflex is the only one that has been found particularly involved in the control of muscle synergies during locomotion (Pierrot-Deseilligny and Burke, 2012), and thus likely constitutes an interesting metric of spinal activity during locomotion in humans, for evaluating the neuromodulation.

In all the subjects, the CP nerve stimulation produced a biphasic facilitation on VL EMG occurring about 30–35 ms after the artifact. Previous studies using the modulations of rectified EMG after CP nerve stimuli reported similar latencies and duration (Marchand-Pauvert and Nielsen, 2002; Iglesias et al., 2008b; Achache et al., 2010). Different methodologies have been used to elucidate the origin of the CPQ reflex, by studying the modulation of averaged EMG, of H reflex amplitude, of the size of motor evoked potential (produced by transcranial magnetic stimulation, TMS), and of single motor unit discharge (Marchand-Pauvert et al., 2005). All the studies converge on the conclusion that the CPQ reflex is mediated by mid-lumbar propriospinal neurons (Pierrot-Deseilligny and Burke, 2012). After stroke, it has been shown that the CPQ reflex is particularly enhanced in patients and might be involved in spasticity (Marque et al., 2001; Maupas et al., 2004), and/or might assist muscle synergies to assist and maintain the upright posture during post-stroke locomotion (Achache et al., 2010). There is no consensus whether the propriospinal hyperactivity after stroke helps or limits the motor recovery after stroke. During walking without sound, the present study also reports an enhanced CPQ reflex in patients, which further supports the spinal hyperexcitability and hyperactivity at the level of the CPQ reflex after stroke. Walking with music, the CPQ reflex returned to normal level in patients, similar to controls. Until then, the CPQ reflex has only been depressed pharmacologically, by antispastics (Rémy-Néris et al., 2003; Maupas et al., 2004). To date, our study is the first one showing that a physiological stimuli (music) can modulate and restore a reflex activity in stroke survivors.

### Possible Mechanisms Underlying the Music-Induced Neuromodulation

The spinal excitations mediated by the propriospinal neurons to lumbar motoneurons are strongly controlled by descending inputs. This was evidenced by studies using TMS, or comparing the excitation at rest and during voluntary movements (Forget et al., 1989a; Marchand-Pauvert et al., 1999, 2005). The descending inputs are likely of cortical origin in part, but also might arise from the extrapyramidal system. Indeed, it has been shown that the CPQ reflex, and the group II component in particular, is depressed by monoamines (Rémy-Néris et al., 2003; Maupas et al., 2004; Marchand-Pauvert et al., 2011), as reported in cats regarding the group II spinal reflexes (Jankowska, 1992; Pierrot-Deseilligny and Burke, 2005). Additionally, the high-frequency deep brain stimulation of subthalamic nuclei in patients with Parkinson disease reduces the CPQ reflex to normal values (Marchand-Pauvert et al., 2011). Therefore, the CPQ reflex is particularly sensitive to a change in the corticospinal inputs and in the neuromodulation from midbrain structures. Accordingly, it has been suggested that the alteration of the descending control to spinal neural networks after stroke, likely contributes to the enhanced propriospinal reflex observed in patients at rest and during locomotion (Marque et al., 2001; Rémy-Néris et al., 2003; Maupas et al., 2004; Achache et al., 2010).

In healthy subjects, TMS over the primary motor cortex inhibits the CPQ reflex during locomotion (Iglesias et al., 2008a), and the cortical contribution to quadriceps activity during walking is weak compared to a more volitional movement like an isolated tonic contraction of quadriceps (Iglesias et al., 2012). Thus, it has been suggested that the control of quadriceps during stabilized locomotion is likely subcortical in origin, and the propriospinal system plays a significant role in the transmission of the motor command to motoneurons, in line with the role of midbrain structures in the control of spinal locomotor central pattern generators (CPGs) in vertebrates (Grillner and El Manira, 2019). Alternatively, the role of proprioceptive feedback, especially muscle spindle group II afferents that are particularly active during TA lengthening contraction at the beginning of stance, has been raised (Marchand-Pauvert and Nielsen, 2002). However, given the weakness in ankle dorsiflexors after stroke and the resulting foot drop, this feedback might be less in patients and likely contributes only poorly to the CPQ reflex. Therefore, we assume that the enhanced propriospinal CPQ reflex during post-stroke walking likely results from modification of the subcortical influence on spinal circuitries likely of reticular origin. In line with this, connections from the cerebral cortex to the reticular formation in the brainstem allow motor commands to be sent over the reticulospinal tract to spinal networks, and after stroke, these pathways are likely particularly involved in the functional recovery (Baker et al., 2015; Li, 2017).

Several studies have reported music-induced excitability changes at the brain level and, given the change in reflex activity observed in resting subjects listening to music, a possible change in descending inputs likely influences the spinal excitability as well (see Introduction). Indeed, it has been proposed that the neuromodulation of spinal reflex as a result of sound exposition, is of reticulospinal origin (startle response; Delwaide and Schepens, 1995). In our study, the music did not produce any startle response and did not influence at all the CPQ reflex in controls; the CPQ reflex was of similar size whether the subjects listened to music or not. Therefore, the possible descending music-induced neuromodulation had only little or no influence on spinal activity during locomotion. One possible explanation would be a saturation of the reticular system during locomotion, but this is unlikely given the possibility of startle during walking. Alternatively, there might be a saturation of the reflex activity under the reticulospinal influence, becoming less sensitive to music-induced additional reticulospinal inputs in controls. Further studies in controls walking at a slower speed (with lower CPQ reflex activity) than their preferred speed (but not as slow as in patients because the CPQ reflex is hardly evoked at ~0.2 m.s^−1^ in controls) would help to determine if music-induced neuromodulation would also occur in controls (Iglesias et al., 2008b; Achache et al., 2010). On the contrary, in stroke patients, the CPQ reflex was depressed by music, reaching normal values as those observed in controls. This might be related to the neuromodulation in the cortico-reticulo-spinal system following stroke, used for motor commands and functional recovery (Baker et al., 2015; Li, 2017). On the other hand, it is not clear whether the reticulospinal hyperexcitability and associated spasticity is a good or maladaptive plasticity (Li, 2017).

### Repercussions on Post-stroke Walking

The results in patients indicate that the cortico-reticulo-spinal system might be modulated after stroke, when using music and sounds as distractors. Indeed, all the patients paid attention to the music during the recording sessions, whether they listened to the melody or AES. The music likely functioned as an external focus of attention that is known to improve the post-stroke walking (Kim et al., 2019). However, the change in spinal excitability was not associated to a change in the biomechanical parameters, while RAS has been shown to improve the cadence and the step length during post-stroke walking (Yoo and Kim, 2016; Magee et al., 2017); walking speed is also enhanced with RAS but since we used the same treadmill velocity (to avoid any change in CPQ reflex due to speed; Iglesias et al., 2008b), we cannot conclude on this parameter. One possible explanation for this result is that we used music with irregular tempo, to avoid any step synchronization, and the absence of change in the gait parameters likely supports the necessity of constant rhythm (like RAS), to observe short term benefits on locomotor cadence. However, it is interesting to note that the arhythmical melody and AES we used were able to modulate the spinal excitability without behavioral repercussion. The patients only performed two short walking sessions with music. There is a possibility that more repetitions are needed to consolidate the plasticity and to observe clear improvements of the walking pattern. In addition, our basic biomechanical approach was too limited and likely not sensitive enough for being really conclusive. Furthermore, we did not investigate the change in walking speed because changing the treadmill speed would have altered the CPQ reflex (Iglesias et al., 2008b); something that needs to be taken into account in future studies.

All the patients preferred the melody compared to AES. We selected the melody because we found it more pleasant than AES, and most of the subjects shared our opinion. The emotional states and their changes are difficult to evaluate but heartbeat and respiratory rhythm are sensitive. However, we did not find any change with music and objectively the subjects did not appear particularly affected in one direction or another by the melody or AES. Therefore, we assume the emotional charge was likely not different between the melody and AES, and they likely paid attention equally to both themes during walking (comparable external focus of attention). On the other hand, they preferred to walk when listening a music or sounds, compared to nothing. Therefore, there is a possibility that the change in spinal excitability might be also related to some extent to the influence of music on mood, as reported at the level of the brain (Altenmüller and Schlaug, 2015). In any case, the present study has shown that listening to music or sounds after stroke is efficient to modulate the synaptic activity at the level of the propriospinal reflex that is known to be particularly involved in locomotion. We assume that repetitive training walking sessions with music may help to counteract the post-stroke spinal hyperexcitability, which limits muscle synergies during walking and hinder to some extent the functional recovery.

## Conclusion

It has been well-established that the rehabilitation with music is more efficient than the one without music (Galinska, 2015). Interestingly, most of the physiotherapists report that a session of rehabilitation is always better with music, the patients performing better than without music but, apparently, they do not know why. On the other hand, our patients reported they preferred walking with music, than without, whatever it was. This study has shown that during only one training session, music and sounds modulate the spinal activity, at the level of the propriospinal network that is known to be particularly involved in stabilized walking. In normal conditions, the CPQ reflex is particularly active when the subjects walk at their preferred speed corresponding to a more automatic locomotion, which does not require too much attention, as compared to slow or fast speed. We discussed the point that music and sounds have likely acted as external focus of attention. Restoring the propriospinal reflex to normal value with music listening might be a sign that the walking control under these conditions was more automatic than without music. This might help recovering locomotor automatisms (with less volitional step control). However, the spinal plasticity was not accompanied by any change of gait parameters, likely because the parameters we tested were not sensitive enough and that more training sessions are required to objectify gait improvement. Future studies using the propriospinal reflex as a biomarker of music-induced plasticity and patient follow-up would be interesting to further confirm the interest of music with or without regular tempo, to improve post-stroke walking.

## Data Availability Statement

All datasets generated for this study are included in the article/Supplementary Material.

## Ethics Statement

The studies involving human participants were reviewed and approved by CPP Ile de France VI—Pitié-Salpêtrière; PHRC 95078, DRCD 070804. The patients/participants provided their written informed consent to participate in this study.

## Author Contributions

IP, CK, PP-D, and VM-P conceptualized the study and has developed the protocol. BH-B, CK, EB, and PP-D have selected the patients and performed the clinical evaluation. VM-P and AL-V have selected the controls. VM-P, BH-B, and AL-V performed the experiments. VM-P and IP analyzed the data and wrote the draft of the manuscript. VM-P performed the statistics. All authors participated in finalizing the manuscript.

### Conflict of Interest

The authors declare that the research was conducted in the absence of any commercial or financial relationships that could be construed as a potential conflict of interest.
